# Time Is Money: The Effect of Mode-of-Thought on Financial Decision-Making

**DOI:** 10.3389/fpsyg.2021.735823

**Published:** 2021-09-27

**Authors:** Lidor Krava, Shahar Ayal, Guy Hochman

**Affiliations:** Baruch Ivcher School of Psychology, Reichman University, Herzliya, Israel

**Keywords:** decision quality, dual-system, information processing style, time pressure, utility maximizing

## Abstract

The dual-system approach holds that deliberative decisions and in-depth evaluation processes lead people to better financial decisions. However, research identifies situations where optimal economic decisions may stem from a more intuitive decision process. In the current work, we present three experimental studies that examined how these two modes-of-thought affect financial decisions. In Study 1, deliberative processes were indeed associated with better one-shot descriptive-based financial decisions. However, Study 2 showed that when participants were asked to make repeated decisions and were required to learn from their experience, the advantage of deliberative over intuitive processes was eliminated. In addition, when participants employed intuitive processes, the quality of their financial decisions improved significantly with experience. Finally, Study 3 showed that the deliberative processing style may lose its advantage when information is not fully available. Overall, these findings suggest that deliberation may contribute to financial decision-making in one-shot decisions. However, when information is lacking, and decisions are repetitive, intuitive processes might be just as good.

## Introduction

People frequently need to make personal and professional decisions in their daily lives. Although some of these decisions can be inconsequential, others, such as financial decisions, can enormously impact individuals’ economic and social lives. These include choosing a savings plan, investing in a pension fund, and taking out a loan. Decision-making models suggest that the best decisions in these situations are made after careful deliberation and a thorough analysis of all the available information (Dijkstra et al., 2013). The importance of deliberate decisions notwithstanding, evidence exists that reasoning or deliberations are not always beneficial to decision quality (e.g., Dijksterhuis et al., 2006; Raoelison et al., 2020).

Advances in technology have changed the face of financial decision-making. The availability of investment tools and online resources enables individuals to base financial decisions on robust logical thinking and analytical analysis of all the available information. At the same time, however, they might expose them to more irrelevant data and marketing traps (e.g., Ayal and Zakay, 2009). For example, a person who wants to select a savings plan might review all the options, consult online resources, and engage in analytical evaluations of financial information. Given the glut of information swamping online resources today, economic decisions can draw on vast amounts of information and require considerable thought to parse (Schwartz, 2004). Here, we argue that financial decisions do not always benefit from a more deliberative process that relies on ample information. Rather, when the decision is complex and involves information that is not easily comprehended (e.g., inflation, compound interest, and disintermediation), knowledge and deliberation do not lead to optimal decisions (Lusardi and Mitchell, 2014). In such cases, financial decisions can benefit from more automatic and frugal processes (Gigerenzer and Todd, 1999; Durbach et al., 2020). For example, Durbach et al. show that intuition can lead to close to optimal portfolio selection.

### The Dual-System Approach: The Intuitive Mode Vs. the Deliberative Mode

According to the dual-system approach (Epstein, 1994; Sloman, 1996; Stanovich and West, 2000; Evans, 2003; Kahneman, 2003), decision-making is based on two distinct cognitive mechanisms (thinking modes). The intuitive system is assumed to be associative, quick, unconscious, effortless, and more error-prone. On the other hand, the deliberative system is characterized by slower, conscious, effortful, and rule-based processes.

Intuitive impressions are generated automatically and can be overridden by conscious, effortful, and deliberative reasoning. Intuitive judgments are thus considered to directly reflect impressions that are not modified by conscious deliberation (Kahneman, 2003; Hogarth, 2005; Evans, 2008). Kahneman and Frederick (2002) argued that erroneous intuitive judgments arise from biased intuitive processes and a lax monitoring of the deliberative system that fails to correct these intuitive violations of normative considerations (c.f., Kahneman, 2003). Thus, people with higher reliance on a deliberative processing style exhibit better decision-making (Kahneman and Frederick, 2002; Kahneman, 2011).

In line with this claim, studies assessing performance on numerical tasks show that analytical rule-based deliberation improves the accuracy and consistency of computations relative to reliance on intuition (McMackin and Slovic, 2000; Beilock and Decaro, 2007; Rusou et al., 2013). Similarly, research finds that people that rely on deliberative processing style are less susceptible to decision biases (e.g., Banks and Oldfield, 2007; Stanovich and West, 2008; Ayal et al., 2012; Kirchler et al., 2017) and that this thinking mode is highly correlated with the Adult Decision-Making Competence scale, a reliable and valid measure of decision quality (Bruine de Bruin et al., 2007, 2012; Bavol’ár and Orosová, 2015).

### The Benefits of the Intuitive Processing Style

These findings notwithstanding, analytical thinking can facilitate the use of irrelevant information and biased behavior (Dijksterhuis and Nordgren, 2006; Ayal and Hochman, 2009). Moreover, research shows no correlation between biased decisions and intuitive thinking style (Ayal et al., 2011, 2012). In a similar vein, intuitive thinking led to more accurate and consistent decisions than analytical one (Bruine de Bruin et al., 2007; Acker, 2008; Glöckner and Herbold, 2011; Usher et al., 2011). For example, in a series of four experiments, Usher et al. (2011) investigated the impact of mindset and distraction manipulations on decision-making quality. The results showed that intuitive strategies could outperform deliberation-analytic strategies for value integration, an operation that is critical in complex decisions. This strengthens the claim that the intuitive mode-of-thought might be superior on some complex decision-making tasks (Usher et al., 2011; Rusou et al., 2013; Brusovansky et al., 2018).

The benefits of intuitive processes were also shown in bargaining situations. For example, acceptance rates of unfair offers in the ultimatum game, which can be justified by rational considerations, increased when participants based their decisions on their gut feelings instead of thoroughly considering the available information (Hochman et al., 2015). These results suggest that in decisions that involve economic (e.g., utility maximization) vs. social (e.g., fairness) considerations, a more economic-rational decision was made when individuals were forced to respond rapidly according to their intuition (for similar conclusions, see Shalvi et al., 2012).

Deliberative thinking tends to engage in active information search during the decision process (Wilson and Schooler, 1991; Kahneman, 2011). However, if irrelevant or complex information is processed deliberatively, the decision may be more biased (Tversky and Kahneman, 1974; Kahneman and Frederick, 2002; Ayal et al., 2011; Hochman et al., 2015). Moreover, intuitive weighting and integrating information that utilizes the most relevant attributes may contribute to the decision and improve its quality (Ayal and Hochman, 2009; see also Dijksterhuis and Nordgren, 2006; Acker, 2008). Therefore, when the available information is incomplete or overly complicated, more intuitive responses can play a crucial role in human decision-making (Damasio et al., 1994; Loewenstein et al., 2001; Greene and Haidt, 2002).

Since many economic decisions require dealing with complex information that is difficult to process and people may lack formal financial knowledge (Lusardi et al., 2010; Lusardi and Mitchell, 2017), a more intuitive process might lead to better decisions (Balasubramnian and Springer, 2020). This is because intuitive processes are based on self-interest and could be less affected by irrelevant information (Shalvi et al., 2012; Hochman et al., 2015). As a result, they might lead to more rational and utility-maximizing decisions. At the same time, a more deliberative process that evaluates more complex and often irrelevant information could lead to more biased decisions (Ayal and Hochman, 2009; Ayal et al., 2011, 2015; Rusou et al., 2013).

Furthermore, experience and familiarity with choice alternatives may ease the negative emotions associated with them (e.g., Zandstra et al., 2000; Yechiam et al., 2019). For example, people reported fewer negative emotions toward objects after repeated exposure to them (Foa and Kozak, 1986; Robinson and Elias, 2005). Similarly, repeated exposure to the same decisions eliminates the negative emotions that reduce risk-taking tendencies and confidence in one’s abilities to make financial decisions (Kuhnen and Knutson, 2011). In turn, this could inhibit people’s intuitive aversion to risky or ambiguous alternatives, especially concerning decisions that involve complicated and unclear terms and concepts (Lusardi et al., 2010; Lusardi and Mitchell, 2017).

The current work presents three studies designed to examine the specific contribution of deliberative and intuitive processing styles to the quality of economic decisions. It explores whether single vs. repeated decisions based on description or experience may hinder the effectiveness of deliberative thinking and lead people to make more biased decisions.

## Study 1: The Effect of Mode-of-Thought on Descriptive-Based Financial Decisions

Stanovich and West (2000) suggested that individual differences play a key role in understanding the disparity between optimal and actual performance. In line with this claim, Zakay and colleagues (Ayal et al., 2011, 2012; Rusou et al., 2013) found that individual differences in cognitive processing styles [indexed by the Rational Experiential Inventory (REI) scale; Pacini and Epstein, 1999] directly influence decision quality (Ayal et al., 2015). In addition, research shows that manipulating information processing style has a marked effect on decision quality (Shiloh et al., 2002; Ayal et al., 2011, 2012; Rusou et al., 2013). In particular, reliance on a deliberative (but not intuitive) thinking style was found to be a crucial predictor of rational behavior (Ayal et al., 2011, 2012).

Study 1 was designed to explore the effect of mode-of-thought manipulation and individual differences in cognitive processing styles on financial decision-making. To do so, we used two one-shot description-based economic scenarios and a lottery choice questionnaire (Holt and Laury, 2002) to assess the quality of participants’ financial judgments. The participants were instructed to make their decisions either quickly and based on their primary gut feelings in the intuitive condition or to take their time and engage in an analytical decision process in the deliberative condition (Usher et al., 2011; Rusou et al., 2013; Hochman et al., 2015).

### Materials and Methods

#### Participants

Ninety-eight Reichman University students (average age=28.15years; SD=10.42; range=20–67; 70 females) took part in this study for a monetary payoff. All participants were native Hebrew speakers. Participants were recruited *via* ads posted in students’ forums. Participation was voluntary and contingent upon signing a consent form. The sample size was predetermined by a power analysis (Faul et al., 2009) to allow 80% power to detect a moderate effect size (95 participants were needed).

#### Design and Procedure

The experimental procedure had three stages. First, participants were randomly assigned to one of two between-subject conditions that manipulated mode-of-thought. Based on Usher et al.’s (2011) “Declared” procedure, mode-of-thought was manipulated by informing participants about the “proven benefits” of decisions that are based on a specific thinking mode. Participants were told that “Research has shown that the best decisions are the ones made using intuition [logic and analytical thought]” and were encouraged to base their preferred choice on their “gut feeling” and general impressions [think carefully and logically about their choices]. We used response time to reinforce the manipulation (Hochman et al., 2015). Participants in the intuitive condition were asked to respond quickly without thinking (in 5s or less). By contrast, in the deliberative condition, participants were asked to respond after carefully considering all the information (for at least 30s). To ensure these instructions were followed, participants in both conditions were told that the computer would monitor their response time and alert the experimenter if they spent too much [too little] time before responding.

Then, participants completed a financial task composed of two blocks. In the first block, three economic scenarios were presented, followed by two or three questions for each scenario to examine the participants’ financial decisions. For example:

“You have decided to purchase a one-month trip package to the US at the cost of $6,000. Your travel agent allows you to purchase the package by credit card in 10 interest-free payments. Your credit card has many pending transactions (although the credit limit has not been exceeded), and you are debating whether to purchase the package. Alternatively, you can take a loan with interest from the bank and buy the package in one full payment. Which option do you prefer?

(A) Use your credit card to make payments.

(B) Use a bank loan to pay everything at once.”

The second block was composed of 10 trials of lottery choices, as developed by Holt and Laury (2002), which measures risk tolerance and utility maximization in decisions under risk. In each trial, participants are asked to choose between two lottery options that differ in risk level and expected value. For instance:

Alternative A:

0.8 chance of getting 19.25 NIS

0.2 chance of getting 0.5 NIS

Alternative B:

0.8 chance of getting 10 NIS

0.2 chance of getting 8 NIS

The choice was made by pressing the button labeled “Alternative A” or “Alternative B” positioned at the top of each description. The dependent variable was the proportion of selections from the alternative yielding the higher expected value [the High-EV option, Option A in the example described above (Yechiam and Hochman, 2013)]. To motivate their choices, participants were told that the computer would randomly select one of the trials at the end of the study, and the chosen trial would be played for an actual payoff. In actuality, all subjects received 10 NIS (approx. 2.5 USD) regardless of their choices.

Finally, participants completed the REI questionnaire (Pacini and Epstein, 1999), a 24 item self-reported inventory that assesses individuals’ tendencies to include analytical and experiential considerations in the decision-making process. The REI consists of two unipolar scales (12 items each), which rank participants on two dimensions of thinking style. The first scale measures engagement and favorability of cognitive activities and corresponds to an analytical (deliberative) thinking style (e.g., I have a logical mind). The second scale measures engagement and favorability of automatic activities and corresponds to an experiential (intuitive) thinking style (e.g., When it comes to trusting people, I can usually rely on my gut feeling). Previous research has shown that the internal consistency reliability coefficient for each scale is high (above 0.85), whereas the correlation between them is small and negligible (Pacini and Epstein, 1999). Thus, the REI is assumed to support Epstein’s (1994) claim of two independent information processing systems. Participants were required to state how true each statement was for them on a scale from 1 (definitely not true of myself) to 5 (definitely true of myself).

The study was designed in VB 6.0. Participants were invited to the lab and were seated comfortably in front of a computer screen. Then, the experimenter provided instructions about the task. Each participant engaged in the task in a private setting. Participants were randomly assigned to either the intuitive mode-of-thought (*n*=50) or the deliberative mode-of-thought (*n*=48) condition. After completing the study, the participants were paid, thanked, and debriefed. The complete materials of this study (as well as the other studies in this manuscript and all the data) can be found at https://osf.io/34duc/. The Ethics Committee of Reichman University approved this study.

### Results and Discussion

#### Preliminary Analyses

Before testing the hypotheses, we assessed the reliability of the REI Hebrew translation. The internal consistency of the REI was adequate for both the Analytical scale (Cronbach’s α=0.83) and the Experiential scale (Cronbach’s α=0.83). In addition, as predicted, the correlation between the two scales was negligible and insignificant (*r*=−0.078, *p*=n.s).

Next, we examined participants’ judgment quality. To do so, each financial decision that maximized utility (i.e., had a higher expected value) was coded “1” (“0” otherwise). For questions with more than two possible alternatives on the economic scenarios task, the normative solution was coded as 1, and the other options were coded as 0. The decision quality was measured as the mean maximization score where a higher score indicates a more economically rational choice. The participants’ judgment quality in the economic scenarios was 0.76 (SD=0.20) and 0.65 (SD=0.19) in the lottery choices.

### Hypothesis Testing

#### Decision-Making Quality in the Economic Scenarios

To test the effect of mode-of-thought on decision-making quality, we calculated the mean EV maximization score for each condition. In the intuitive condition (*n*=50), the mean score was 0.71 (SD=0.20), and 0.81 (SD=0.18) in the deliberative condition (*n*=48). An independent samples *t*-test revealed a significant difference between the two conditions (t(96)=−2.49, *p*<0.05). Thus, participants who were encouraged to use in-depth evaluation processes exhibited more economically rational choices than participants who were instructed to employ intuitive processes.

A multiple (stepwise) regression analysis was conducted to examine the effect of mode-of-thought manipulation (deliberative mode=1), individual differences in the two processing styles, and their interaction term on decision-making quality. The predictors were entered into the model in a single block using the stepwise method (with a probability for F of *p*=0.10 as the exclusion criterion and *p*=0.08 as the inclusion criterion). The results indicated that the best model included the interaction between the analytical and experiential scales (*β*=0.24, *p*=0.017) and the mode-of-thought manipulation (*β*=0.19, *p*=0.052; *F*(2, 95)=6.22, *p*<0.01, *R*^2^=11.6%). The analytical (*t*=0.022, *p*=0.982) and experiential (*t*=0.176, *p*=0.861) scales were not significant predictors. The results of this analysis are summarized in Table 1.

**Table 1:** Relationship between mode-of-thought manipulation, information processing styles (REI), and the decision quality in the economic scenarios.

Variables	Regression model		
	*β* (standardized coefficients)	*t*	value of *p*
Mode-of-thought	0.19	1.965	0.052
Analytical^*^Experiential scale	0.24	2.427	0.017
**Model fit**			
R^2^	0.116		
F	6.225^**^		
DF	2,95		

*N=98*,

** *p<0.01*.

Since the deliberative mode-of-thought was coded as 1, these results suggest that greater deliberative considerations resulted in more rational decisions on this task. However, in line with Ayal et al. (2011), the interaction between the analytical and experiential scales suggests that participants who rely on both processes are less prone to judgmental biases.

To examine the effect of repetition, we tested the participants’ performance over time. A 6 (question order – within-subject variable)×2 (mode-of-thought manipulation – between-subject variable) repeated measures ANOVA was conducted to examine decision quality. This analysis revealed a significant main effect for order [*F* (5, 92)=5.98*, p<0.001*], indicating that all the participants improved over time. In addition, there was a main effect for mode-of-thought [*F*(1,96)=6.24, *p*<0.05], as well as a marginally significant order × mode-of-thought interaction [*F* (5,92)=2.31, *p=0*.051]. This pattern of results indicates that participants in the deliberative condition exhibited more economically rational choices than participants in the intuitive condition. However, the quality of the decisions improved with time in both modes-of-thought (albeit more rapidly in the deliberative one).

#### Decision-Making Quality on the Lottery Task

To examine judgment quality on the lottery (Holt and Laury, 2002) task, the proportion of selecting the alternative yielding the higher expected value was calculated for each participant (Yechiam and Hochman, 2013). In the deliberative condition, the average rational choice was 0.74 (SD=0.17), compared to 0.56 (SD=0.16) in the intuitive condition. An independent samples *t*-test revealed that this difference was significant (t(96)=−5.02, *p*<0.001). Thus, the deliberative mode-of-thought led to more economically rational choices than the intuitive mode.

A multiple (stepwise method) regression analysis was conducted to examine the effect of mode-of-thought and processing style on decision quality on the lottery task. The results showed that the best model included the mode-of-thought manipulation (*β*=0.43, *p*<0.001) and the analytical scale (*β*=0.3, *p*=0.002; *F*(2,95)=18.6, *p*<0.001, *R*^2^=28.1%). These results are summarized in Table 2. The experiential scale was not significant (*p*=0.77). Thus, similar to the previous findings, the results suggested that in-depth considerations resulted in more rational decisions (in terms of EV) than intuitive responses. In line with previous research (Ayal et al., 2011, 2012, 2015), there was a positive correlation between the analytical scale and decision quality, but at the same time, no negative connection between rational decision-making and experiential thinking style.

**Table 2:** Relationship between mode-of-thought manipulation, information processing styles (REI), and the decision quality in the lottery task.

Variables	Regression model		
	*β* (standardized coefficients)	*t*	value of *p*
Mode-of-thought	0.43	4.99	0.0001
Analytical scale	0.3	3.11	0.002
**Model fit**			
R^2^	0.281		
F	18.6^***^		
DF	2,95		

*N=98*,

*** *p<0.001*.

Study 1 reveals the clear benefit of the deliberative mode-of-thought in making financial decisions. While decision quality increased as participants answered more questions on the economic scenarios, this effect was more pronounced for the deliberative mode-of-thought. This pattern of results is consistent with the dual-system approach, which holds that rational behavior stems from a deliberative thought process (e.g., Kahneman, 2003, 2011). Nevertheless, as in previous studies (Ayal et al., 2011, 2012, 2015), we found no correlation between decision quality and experiential thinking style. In addition, the interaction between the analytical and experiential scales was a better predictor of rational choice behavior (Ayal et al., 2011, 2012). In line with Ayal et al.’s (2011) claim, these results suggest that deliberative thinking may benefit from the additional input of the intuitive system in certain situations. Thus, the combination of highly deliberative and intuitive considerations seems like an important factor in normative reasoning.

## Study 2A: Repeated Financial Decisions

Experience and familiarity with choice alternatives ease negative emotions (Zandstra et al., 2000; Yechiam et al., 2019) and lead people to make better financial decisions. Hence, in Study 2a, we examined whether repeated financial decisions would benefit from thorough deliberative considerations, relative to intuitive ones. To do so, decision quality was assessed in two tasks: a modified version of the debt management game with repeated trials (Amar et al., 2011) and the Adherence to Biased Judgments questionnaire (Ayal et al., 2011).

### Materials and Methods

#### Participants

One hundred Israelis took part in the study. Forty-four were males, and 49 were females (seven participants did not indicate their gender). The average age was 39.4years (SD=13.73; range=20–74). All participants were native Hebrew speakers. Participants were recruited through posts on social media. Participation was voluntary and contingent upon signing a consent form. The sample size was predetermined by a power analysis (Faul et al., 2009) to allow 80% power to detect a moderate effect size (based on the effect size of Study 1, 94 participants were needed).

#### Design

The first stage of the experiment consisted of the mode-of-thought manipulation, as in Study 1. Next, participants were presented with a Qualtrics web-based questionnaire composed of two blocks. The first block was a modified version of the debt management game (Amar et al., 2011). In this game, participants are given multiple debt accounts that vary in amounts and annual interest rates. The game has a total of six rounds. At the beginning of each round, participants receive a hypothetical sum of money they can use as a down payment on one of the two open debt accounts. Each round presents a pair of different debts. For instance,

“Given a monetary amount of 1000 NIS, which of the two debts do you prefer to (partially) repay with this money?

Debt A: Initial Amount 3000-, NIS; Annual Interest Rate 2.5%, OR

Debt B: Initial Amount 8000-, NIS; Annual Interest Rate 2%?”

The first round has Debt 1 vs. 2, the second round has Debt 2 vs. 3, and so on. All six rounds were presented in the same fashion to all participants. In each round, the participants were asked to choose the debt they wanted to reduce by allocating their money. A financially optimal player should allocate the available sum to the open debt with the highest interest rate in each round. By contrast, a non-rational player would focus on the amount and allocate the available money to the smallest open debt to further reduce it (“debt account aversion”; Amar et al., 2011). The debt management game is a financial task that includes complex information that is difficult to process (such as interest rate, the debt amount, and the available money). This study aimed to assess potential differences in choice quality motivated by deliberative vs. intuitive considerations.

The second block was composed of four prototypical questions (the Bias Thinking questionnaire adapted from Ayal et al., 2011) that measure adherence to biased judgments. Specifically, Question 1 taps irrational diversification (Ayal and Zakay, 2009), i.e., a false evaluation of diversity based on the perceived rather than the normative value. Question 2 examines the ratio bias, i.e., the tendency to judge a low probability event as more likely when presented as a ratio with large numbers than a ratio with small numbers (e.g., 9/100 vs. 1/10; Kirkpatrick and Epstein, 1992). Question 3 tests the availability heuristic, i.e., individuals’ tendency to estimate the frequency of an event “by the ease with which instances or associations come to mind” (Tversky and Kahneman, 1973, p.208). Finally, Question 4 examines the conjunction fallacy (Tversky and Kahneman, 1983), i.e., people’s tendency to ignore the statistical cut-off law and estimate that the probability of the conjunction between two events together is greater than the probability of a single event alone. The order of presentation was identical for all participants.

#### Procedure

A link to the experimental questionnaire was sent to participants *via* email. Participants were randomly assigned to either the intuitive (*n*=58) or the deliberative mode-of-thought (*n*=42) condition. The study lasted about 10min. After completing the questionnaire, the participants were debriefed and were given the experimenter’s contact information for any questions. The Ethics Committee at Reichman University approved this study.

### Results and Discussion

To assess decision quality, each choice predicted by the normative solution was coded “1,” and each response predicted by the corresponding bias was coded “0.” Similarly, each financial decision that was based on the optimal solution was coded “1” (and “0” otherwise). Thus, the decision quality was measured by calculating the mean of each subject’s choices, with a higher score indicating a more rational choice.

**Figure 1:**
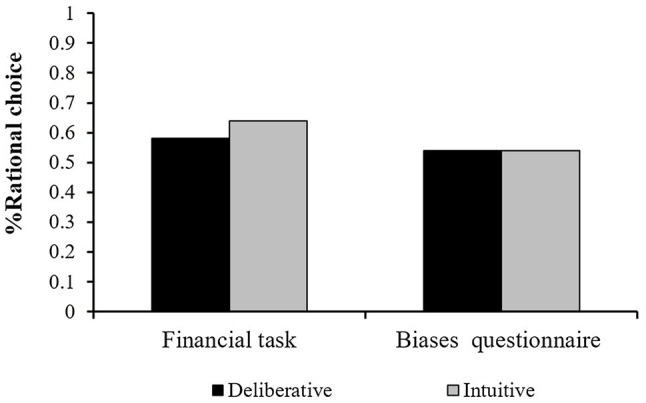
Mean Decision quality (% of rational choice) in Study 2a’s tasks as a function of mode-of-thought manipulation.

First, we examined decision quality on each block (the financial task and the biases questionnaire) as a function of the experimental condition (mode-of-thought). No difference was found between conditions for the biases questionnaire. That is, the participants’ mean score for the rational choice was 0.54 in the intuitive (SD=0.25) and 0.54 (SD=0.27) in the deliberative conditions (t(98)=0.01, n.s.). There was no difference even when we compared each question on the biases questionnaire separately. Similarly, the mean rational score on the financial task was 0.64 (SD=0.27) in the intuitive condition vs. 0.58 (SD=0.26) in the deliberative condition. An independent samples *t*-test revealed that this difference was not significant (t(98)=−1.1, n.s.). These results are presented in Figure 1. Overall, this pattern of results suggests that in financial decisions with repetition, a deliberative processing evaluation that places considerable strain on the cognitive system does not outperform the intuitive mode-of-thought.

**Figure 2:**
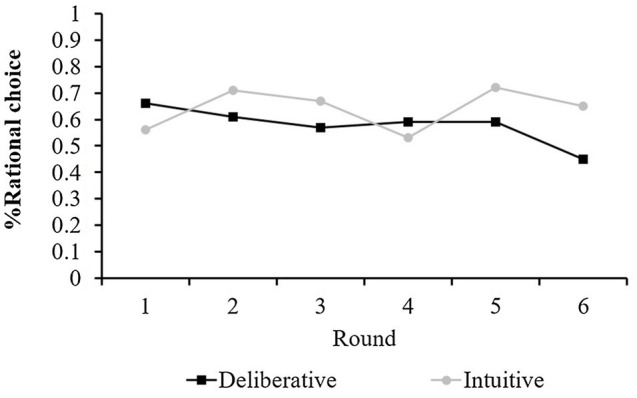
Mean Decision quality (% of rational choice) in Study 2a for each of the six rounds of the financial task as a function of mode-of-thought manipulation.

Since the financial task consisted of six rounds, we further tested the participants’ performance over time. A 6 (round as a within-subject variable)×2 (mode-of-thought as a between-subject variable) repeated measures ANOVA was conducted to examine decision quality. These results are illustrated in Figure 2. No main effect was found for mode-of-thought [*F* (1, 98)=1.22, n.s.] or for block [*F*(5, 94)=0.96, n.s.]. However, a significant round × mode-of-thought interaction was observed [*F*(5,94)=2.51, *p*<0.05], suggesting a significant improvement in decision quality with time under the intuitive condition, but not in the deliberative one. Post-hoc analyses revealed a significant difference in favor of intuitive thinking only on the final trial (t(98)=−2.04, *p*<0.05); however, no difference between the mode-of-thought conditions was found on the other trials. This pattern of results supports previous research (Ayal et al., 2011; Usher et al., 2011), as well as our theoretical claim that intuitive judgments may be more beneficial for financial decisions that require repetition.

## Study 2B: The Debt Management Game

Study 2b examined the robustness of the findings of Study 2a. This study used the same debt management game (Amar et al., 2011) as in Study 2a with minor changes (detailed below). Since we found in Study 1 that deliberative processes are strong contributors to decision quality, we also examined whether individual differences in processing styles (measured on the REI scale) would affect the quality of the financial decision.

### Materials and Methods

#### Participants

One hundred and thirty-six Israelis took part in the study. Seventy-one were males, and 65 were females. The average age was 41.43years (SD=14; range=21–69). All participants were native Hebrew speakers. These participants also took part in Study 3. Participants were recruited through posts on social media. Participation was voluntary and contingent upon signing a consent form. The sample size was predetermined using a power analysis (Faul et al., 2009) to allow 80% power to detect a moderate effect size similar to Study 1 (94 participants were needed).

#### Design

Participants were presented with a Qualtrics web-based questionnaire. First, participants completed the REI questionnaire (Pacini and Epstein, 1999), as in Study 1. Then, participants were presented with the mode-of-thought manipulation (deliberative vs. intuitive mode-of-thought) as in the previous studies. Finally, they were asked to complete the same debt management game (Amar et al., 2011) as in Study 2a, except that the available amount of money given to participants at the beginning of each round was enough to eliminate the debts, not just reduce them. The game had seven rounds, which were presented in the same order for all participants.

#### Procedure

A link to the experimental questionnaire was sent to participants *via* email. Participants were randomly assigned to one of two between-subject conditions [intuitive mode-of-thought (*n*=70) and deliberative mode-of-thought (*n*=66)]. After completing the questionnaire, the participants were debriefed and were sent the link to Study 3. The Ethics Committee at Reichman University approved this study.

### Results and Discussion

#### Preliminary Analysis and Manipulation Check

The reliability of the REI scale indicated high internal consistency for the analytical (Cronbach’s α=0.84) and the experiential (Cronbach’s α=0.87) scale. In addition, as predicted, no correlation was found between the two scales (*r*=0.102, *p*=0.24).

Next, we examined the mean response time in the two experimental conditions (mode-of-thought manipulation). The mean response time was 9.34m (SD=3.27) in the time-pressure condition (i.e., intuitive mode-of-thought) and 27m (SD=14.63) in the no-time-pressure condition (i.e., deliberative mode-of-though). An independent samples *t*-test revealed that this difference was highly significant [t(71.14)=9.578, *p<0.0001*]. These results validated our manipulation and suggested that the participants understood and adhered to the instructions to follow their gut feelings or examine all the available information (depending on the experimental condition; Hochman et al., 2015).

Each financial decision that was based on the utility maximizing principle was coded “1” (and “0” otherwise). The decision quality was measured by calculating the mean of each participant’s choices. Across participants and conditions, the mean score for rational choice in the debt management game was 0.65 (SD=0.23).

#### Decision Quality in the Debt Management Game

The mean rational choice score in the debt management game was 0.67 (SD=0.22) in the intuitive condition, compared to 0.63 (SD=0.24) in the deliberative one. As in Study 2a, this pattern of results hinted at a slight advantage (of 4%) for intuitive processes relative to deliberative ones. However, an independent samples *t*-test revealed that this difference was not significant (t (134)=−1.039, *p*=0.302).

**Figure 3:**
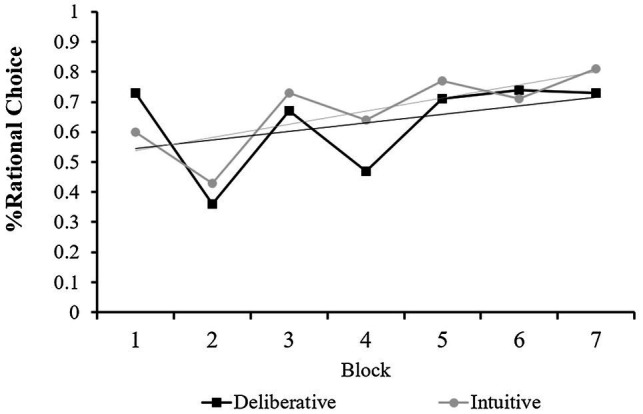
Mean Decision quality (% of rational choice) in Study 2b for each of the seven rounds of the debt management game as a function of mode-of-thought manipulation.

As in the previous studies, we also tested the participants’ performance over time. A 7×2 repeated measures ANOVA was conducted to predict decision quality with block (1 to 7) as a within-subject factor and mode-of-thought (intuitive vs. deliberative) as a between-subjects factor. This analysis revealed a significant main effect for block number [*F* (6, 129)=7.02, *p*<0.0001], and a marginally significant block × mode-of-thought interaction [*F*(6,129)=2.06, *p*=0.06]. By contrast, no effect was found for mode-of-thought [*F* (1, 134)=1.08, *p*=0.301, n.s.]. These results are presented in Figure 3 and show a significant improvement in decision quality with time in both conditions. However, there was more improvement in the intuitive condition than in the deliberative one. Thus, similar to Study 2a, repeated experience with financial decisions improved decision quality, and this effect was more pronounced in the intuitive condition.

We conducted a multiple stepwise regression analysis to test the relationship between mode-of-thought manipulation, information processing style, and decision quality. The results showed that the best model only included the analytical scale (*β*=0.3, *p*=0.0003; *F*(1,134)=13.6, *p*<0.001, *R*^2^=9.3%). These results are summarized in Table 3. The experiential scale was not significant (*p*=0.73). Thus, consistent with previous research, the analytical scale was a crucial predictor in optimal decision-making (Ayal et al., 2011, 2012).

**Table 3:** Relationship between mode-of-thought manipulation, information processing styles (REI), and decision quality as the dependent variable.

Variables	Regression model		
	*β* (standardized coefficients)	*t*	Significance
Analytical scale	0.3	3.7	0.0003
**Model fit**			
R^2^	0.093		
F	13.6^***^		
DF	1,134		

*N=136*,

*** *p<0.001*.

Overall, Studies 2a and 2b provide converging evidence that deliberative considerations do not outperform automatic evaluations under situations of repeated financial selections. In the debt management game, participants needed to select the account with the highest interest rate rather than the lowest nominal value (Amar et al., 2011). Both studies showed that the ability to identify the correct debt to repay improved significantly with experience. Importantly, there was greater improvement when participants based their decisions on intuition rather than deliberation. In addition, similar to previous findings (Ayal et al., 2011; Usher et al., 2011), no difference was found for biased decisions between deliberative and intuitive mode-of-thought. These findings extend previous research showing that intuitive reasoning may be associated with highly normative judgments (Davis et al., 1993; Glöckner, 2007; Ayal et al., 2011).

Our results suggest that deliberative processes that include irrelevant information (e.g., the number of open debts) might impede decisions (Ayal et al., 2011). At the same time, intuitive processes might be less affected by it, as they are more oriented toward self-interest (Hochman et al., 2015). Furthermore, experience and familiarity with choice alternatives which ease negative emotions (Zandstra et al., 2000; Yechiam et al., 2019) lead people to make better financial decisions. However, these studies focused on description-based decisions, in which all information is provided. Study 3 focused on repeated financial decisions that could be learned from experience to examine the effect of mode-of-thought on decision quality in this kind of situational condition (i.e., learning from experience).

## Study 3: Learning From Experience

The goal of Study 3 was to shed more light on the interplay between mode-of-thought and decision quality in repeated financial decisions. Specifically, we examined how learning and experience with a task influence decision quality under deliberative vs. intuitive mode-of-thought conditions. To do so, we used a modified version of the Repeated Investment Task (adapted from Thaler et al., 1997). This repeated-trials investment task is composed of 20 choices administered in either a full or a partial feedback condition. The task requires people to learn from their own experience which of two investment plans is optimal. This served to examine whether the repetition of financial decisions and learning benefit from deliberative or intuitive processes.

### Materials and Methods

#### Participants

One hundred and thirty-six Israelis participated in the study (65 women). The sample size was predetermined by a power analysis (Faul et al., 2009) to allow 80% power to detect a moderate size effect (112 participants were needed). Study 3 was administered directly after Study 2b to the same participants.

#### Design

Participants were presented with a Qualtrics web-based questionnaire composed of two blocks. In the first block, participants filled in the REI questionnaire (Pacini and Epstein, 1999) as in Study 1. Next, participants were presented with the mode-of-thought manipulation of the previous studies (deliberative vs. intuitive mode-of-thought).

The final block consisted of a repeated saving task adapted from Thaler et al. (1997). Subjects were required to manage a small savings plan in the amount of 20,000 NIS for a five-year period. Each year involved four trials, which represented quarters. Thus, the entire task comprised 20 trials in total. The bank clerk suggested two different savings plans (Plan A and Plan B): Plan A with a variable interest of prime plus 1.5% and plan B with a fixed interest of 2.5%. Each quarter, participants were allowed to switch plans or to stick with the previously selected plan. According to the rational model, optimal players should allocate their money to the plan with the highest interest rate on each trial, since this plan would yield the highest expected return. Plan A had high variance but also high-profit expectancy vs. Plan B, which was more secure but with a lower profit expectancy. Participants had to learn about the risks and returns of the two plans through experience (Thaler et al., 1997).

Participants were randomly assigned to one of two feedback conditions, full or partial. In the full feedback condition, participants received information about the “prime” interest rate at the end of each quarter (savings period). In the partial feedback condition, the prime rate information was only provided on every other trial, starting from Trial 1. This served to examine participants’ financial preferences over time and whether learning from experience (under full and partial feedback) affected their preferences.

#### Procedure

A link to the experimental questionnaire was sent to participants *via* email. Participants were randomly assigned to one of four between-subject conditions: intuitive mode-of-thought with full feedback (*n*=31), deliberative mode-of-thought with full feedback (*n*=37), intuitive mode-of-thought with partial feedback (*n*=39), and deliberative mode-of-thought with partial feedback (*n*=29). The study lasted approximately 20min. After completing the questionnaire, the participants were debriefed and were given the experimenter’s contact information for any questions. The Ethics Committee at Reichman University approved this study.

### Results and Discussion

As in the previous studies, each response that was based on utility maximization was coded as “1” (“0” otherwise). The decision quality was measured by calculating the mean of each subject’s choices. The closer the score to 1.0, the more rational the participant was considered (in terms of EV maximization). Across participants and conditions, the mean score for rational choice in the repeated saving task was 0.59 (SD=0.25).

#### Decision Quality in the Repeated Savings Task

**Figure 4:**
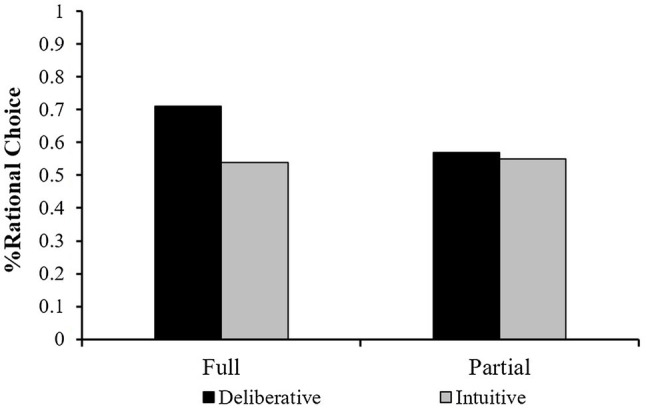
Mean Decision quality (% of rational choice) in Study 3 as a function of feedback (Full vs. Partial) and mode-of-thought manipulation.

To test the effect of mode-of-thought and feedback on decision quality on the savings task, we calculated the mean rational choice for each condition. This analysis revealed that in the intuitive condition, the mean score was 0.54 (SD=0.26) under full feedback and 0.56 (SD=0.21) under partial feedback. In the deliberative condition, the mean score was 0.71 (SD=0.25) under full feedback and 0.57 (SD=0.25) under partial feedback. These results are presented in Figure 4.

A two-way ANOVA revealed a significant effect for the mode-of-thought condition [*F* (1,132)=4.72, *p*<0.05] and a marginally significant interaction effect [*F*(1,132)=3.29, *p*=0.072]. No main effect was found for feedback [F (1,132)=1.68, *p*=0.197, n.s.]. This pattern of results suggests that asking participants to make decisions based on deliberative processes resulted in higher financial decision quality than the intuitive responses. However, the deliberative thinking scores were only noticeably better in the full feedback condition.

#### Moderation Analyses

We conducted a moderation analysis to examine whether the analytical information processing style moderated the effect of feedback (full vs. partial) on decision quality in the repeated savings task. Feedback (as a binary variable, full=1; partial=2) and the analytical scale (categorical variable based on the median split; low coded as 0 and high coded as 1) were simultaneously entered as predictor variables. Following Aiken and West (1990), the predictor variables were centered before conducting the regression analyses. All analyses were performed using SPSS20 with the PROCESS macro (Hayes, 2012) to address the moderation hypotheses.

The analysis revealed a significant marginal main effect for the analytical thinking style (*β*=0.15, B=0.07, *t*=1.78, *p=0.07*), indicating that high analytical participants made better decisions in the repeated savings task. In addition, the interaction between feedback and the analytical scale was significant (*β*=0.17, B=0.17, *t*=2.01, *p=0.04*). No main effect for the feedback condition was found (*β*=−0.13, B=−0.06, *t*=−1.6, *p=0.11,* n.s.).

**Figure 5:**
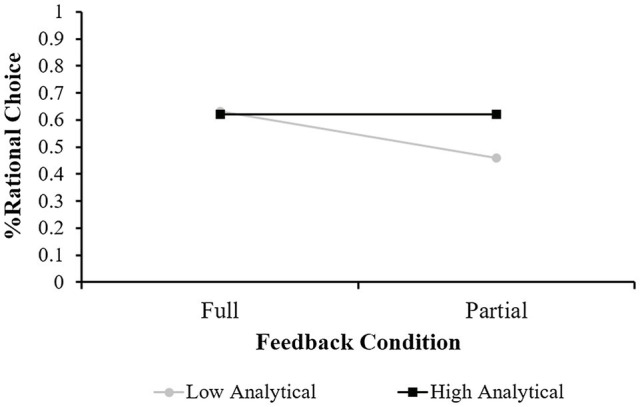
Mean Decision quality (% of rational choice) in Study 3 as a function of feedback condition for people high and low in the analytical scale of the REI.

To understand the nature of this interaction effect, we followed Aiken and West’s procedure (1990) and estimated two regression lines of feedback condition on decision quality at two levels (high and low) of the analytical scale. Simple slope tests indicated that the feedback condition was only significantly associated with financial decision quality among participants low on the analytical scale (*β*=−0.34, B=−0.17, *t*=−2.57, *p<0.05*). This interaction effect is presented in Figure 5.

As shown in the figure, there was no difference in the decision quality between the high and low analytical participants in the full feedback condition. However, high analytical participants were more rational than low analytical participants under the partial feedback condition. That is, in cases where the information was not presented to the decision-maker in full, not relying on analytical thinking may have hampered the quality of the participants’ financial decisions.

As in Study 1, the results of Study 3 point to a clear advantage for deliberative processes in financial decision-making when all the information required for the decision is presented to the decision-maker. In contrast, there was no difference in decision quality between the deliberative and intuitive modes-of-thought conditions when information was lacking. Moreover, individual differences in the analytical style did not influence decision quality when full feedback was provided to the participants. However, when information was partial, low analytical participants were more prone to biased economic decisions. Thus, low analytical thinking (but not high experiential one) may impede rationality in situations where full information is unavailable.

## General Discussion

Models of human reasoning suggest an integrative approach which posits that human decision-making is a product of an intuitive and a deliberative system (Epstein, 1994; Sloman, 1996; Stanovich and West, 2000; Evans, 2003). Both systems play an important role in determining decision quality. However, under certain conditions, each system can lead to optimal decisions (Ayal et al., 2011, 2015; Usher et al., 2011; Shalvi et al., 2012; Hochman et al., 2015). The present study further probed the specific contribution of deliberative and intuitive processes to the quality of economic decisions (Alós-Ferrer and Strack, 2014).

Study 1 confirmed that a deliberative mode-of-thought leads to better financial decisions. At the same time, however, intuitive processes and their interaction with deliberative ones contribute to rational choice behavior (Ayal et al., 2011, 2012). Studies 2a and 2b showed that the quality of debt allocation decisions improved significantly with experience. This improvement was more pronounced when participants based their decisions on intuition rather than deliberation. These findings provide converging evidence that deliberative financial decisions can be ineffective and may impair decision quality under repeated selections. The results of the debt management game further suggest that decisions that are considered economically rational might be more automatic, and secondary considerations that are more psychological in nature could focus people’s attention on irrelevant data and thus lead to sub-optimal financial decisions (Hochman et al., 2015).

In line with Study 1, Study 3 showed that deliberative processes led to better financial decision-making when all the information needed for the decision was available. In contrast, when information was partial, no difference in decision quality was found between the two systems. Moreover, low analytical participants were more prone to biased economic decisions when information was lacking. Thus, deliberative thinking may be crucial for rationality in complex real-life situations where experience-based information is not easily retrieved. Taken together, these three studies highlight conditions under which deliberation and thorough considerations may contribute to financial decision-making.

Rationality is defined as adherence to the normative solution or preference for options that provide the highest expected utility (Simon, 1955). Presumably, as long as the decision at hand is simple, straightforward, and does not require complex processing, deliberate thinking can outperform intuitive thinking and lead to better choices (Ayal and Hochman, 2009). In description-based decisions, all the information is available to the decision-maker. Thus, the deliberative system engages in active information search, which leads to selecting more relevant attributes during the decision process (Wilson and Schooler, 1991). This results in a more rational decision. However, in situations where irrelevant or complex information is processed deliberatively, this may lead to more rather than less biased thinking (Tversky and Kahneman, 1974; Kahneman and Frederick, 2002; Ayal et al., 2011; Hochman et al., 2015). This may occur because the decision is complex and requires information that is not easily accessible. Thus, people tend to rely on accessible rather than relevant information. As shown here, this attribute substitution may occur more frequently in deliberative judgments than intuitive ones, contrary to what was previously assumed (Kahneman and Frederick, 2002).

Importantly, the current research is only a first step toward a more comprehensive understanding of the relationship between information processing style and the quality of financial decisions. Future research should identify more conditions in which deliberative processes no longer improve economic decisions.

### Limitations and Future Directions

Except for Study 1, our studies were not incentivized, and participants were asked to engage in hypothetical financial tasks without an actual incentive structure. However, the results support previous findings (Davis et al., 1993; Glöckner, 2007; Ayal et al., 2011, 2015) and highlight situations in which intuitive thinking leads to well-adjusted judgments. Future research should assess whether similar results would be obtained in decisions that have actual monetary payoffs to examine the generalizability of our results to more “natural” decisions. In addition, in all three studies, we focused on the difference between repeated decisions (e.g., the debt management game and savings tasks) and one-time decisions (e.g., economic scenarios). Therefore, other factors that may lead to intuitive decision-making should be examined in future research; for instance, the effect of framing economic problems in terms of loss or gain (Tversky and Kahneman, 1981) on the relationship between thinking mode and financial decision-making quality. Future research could also explore individual differences, such as financial literacy, age, gender, and other demographics, which may affect the relationship between thinking mode and performance on financial tasks. Finally, Studies 2b and 3 were conducted sequentially with the same sample. Therefore, future research should examine whether the same results would be obtained with different samples.

### Concluding Remarks

Our results suggest that in complex situations, reliance on deliberative processes (particularly for people low in analytical thinking) may impair decision quality due to the weighing of irrelevant information. Thus, when the decision is not overly important and does not require formal knowledge, intuitive processes might benefit the decision-maker (Rusou et al., 2013; Ayal et al., 2015) and save valuable time (Li et al., 2016). Further research and a better understanding of the conditions that enable good economic decisions will help people without a financial background or a good grasp of economics to make better real-life financial decisions.

## Data Availability Statement

The datasets presented in this study can be found in online repositories. The names of the repository/repositories and accession number(s) can be found in the article/Supplementary Material.

## Ethics Statement

The studies involving human participants were reviewed and approved by the School of Psychology at Reichman University review board. The patients/participants provided their written informed consent to participate in this study.

## Author Contributions

LK and GH contributed to the conception of the study and wrote the first draft of the manuscript. LK, SA, and GH contributed to the design. LK collected the data and organized the database. GH and SA performed the statistical analysis. All authors contributed to manuscript revision, read, and approved the submitted version.

## Conflict of Interest

The authors declare that the research was conducted in the absence of any commercial or financial relationships that could be construed as a potential conflict of interest.

## Publisher’s Note

All claims expressed in this article are solely those of the authors and do not necessarily represent those of their affiliated organizations, or those of the publisher, the editors and the reviewers. Any product that may be evaluated in this article, or claim that may be made by its manufacturer, is not guaranteed or endorsed by the publisher.
