# Supplementary material for: Time Is Money: The Effect of Mode-of-Thought on Financial Decision-Making
Source: Front Psychol. 2021 Sep 27;12:735823. doi: 10.3389/fpsyg.2021.735823 (PMC8503517; doi:10.3389/fpsyg.2021.735823)
Supplement: Supplementary file 1 [file Data_Sheet_1.PDF]

Appendices

Appendix A: Informed Consent Form

Dear participant,

This study deals with judgment and decision-making. It takes about 10 minutes to complete. Participation is completely anonymous. The data will only be marked by a serial number without any identifying information. The data will be used solely for the purposes of the present study. Participation in the study is voluntary. You can refuse to participate in this experiment or withdraw at any time with no penalties.

For any questions or comments on the experiment, please contact the researcher at :

Lidor - 054-XXXXXXX

By signing this consent form, you confirm that you understand the information above and agree to participate in the experiment.

Thank you for your participation.

Appendix B: Demographic Questionnaire

Demographic Questionnaire

Please fill in the following details. The information will solely be used for this study and is confidential.

1. Gender: Male/Female
2. Age:____________

Appendix C: The Debt Management Game (adapted from Amar, Ariely, Ayal, Cryder, & Rick, 2011).

For purposes of this publication, the optimal choice in each round is underlined.

Table 1 – The Interest Rate and Initial Size of Each Debt

	Initial Amount (NIS)	*Annual Interest Rate (%)*
Debt 1	3,000	2.50
Debt 2	8,000	2.00
Debt 3	11,000	3.50
Debt 4	13,000	3.25
Debt 5	52,000	3.75
Debt 6	60,000	4.00

Round 1

**Monetary Amount (NIS) 1000**		
	**Debt B**	**Debt A**
**Initial Amount (NIS)**	-8000	-3000
**Annual Interest Rate (%)**	2	2.5
**Which of the two debts do you prefer to [partially] repay by investing the amount you received in this round?**<br>Debt A Debt B		

Round 2

**Monetary Amount (NIS) 2000**		
	**Debt B**	**Debt A**
**Initial Amount (NIS)**	-11,000	-8,000
**Annual Interest Rate (%)**	3.5	2
**Which of the two debts do you prefer to [partially] repay by investing the amount you received in this round**? Debt A Debt B		

Round 3

**Monetary Amount (NIS) 3500**		
	**Debt B**	**Debt A**
**Initial Amount (NIS)**	-13,000	-11,000
**Annual Interest Rate (%)**	3.25	3.5
**Debt** A Debt B		

Round 4

**Monetary Amount (NIS) 5000**		
	**Debt B**	**Debt A**
**Initial Amount (NIS)**	-52,000	-13,000
**Annual Interest Rate (%)**	3.75	3.25
**Which of the two debts do you prefer to [partially] repay by investing the amount you received in this round** ? Debt A Debt B		

Round 5

**Monetary Amount (NIS) 6500**		
	**Debt B**	**Debt A**
**Initial Amount (NIS)**	-60,000	-52,000
**Annual Interest Rate (%)**	4	3.75
**Which of the two debts do you prefer to [partially] repay by investing the amount you received in this round?**<br>Debt A Debt B		

Round 6

**Monetary Amount (NIS) 7000**		
	**Debt B**	**Debt A**
**Initial Amount (NIS)**	-8,000	-60,000
**Annual Interest Rate (%)**	2	4
**Which of the two debts do you prefer to [partially] repay by investing the amount you received in this round?**<br>Debt A Debt B		

Appendix D: Bias Thinking Questionnaire (adapted from Ayal et al., 2011)

(for purposes of this article, the rational response is underlined)

Question 1 – Irrational diversification (Ayal & Zakay, 2009).

Biased response: Choosing the lottery with the higher perceived diversity but lower chances of winning:

Imagine you purchased an expensive LCD TV at a popular shopping site. The site then offers you a choice to enter one of two lotteries. Which lottery do you prefer?

1. <u>Lottery A – A raffle of one car (Volvo S80) out of 10,000 consumers</u>
2. Lottery B – A raffle of 9 cars (3 Volvo S80, 3 Toyota Corolla, and 3 Honda Civic) out of 100,000 consumers

Question 2 - Ratio bias (Denes-Raj, Epstein and Cole, 1995)

Biased response: Preferring to draw a marble from the large urn (e.g., 9 out of 100) over the small urn (e.g., 1 out of 10):

Consider these 2 urns filled with marbles

Urn A Urn B

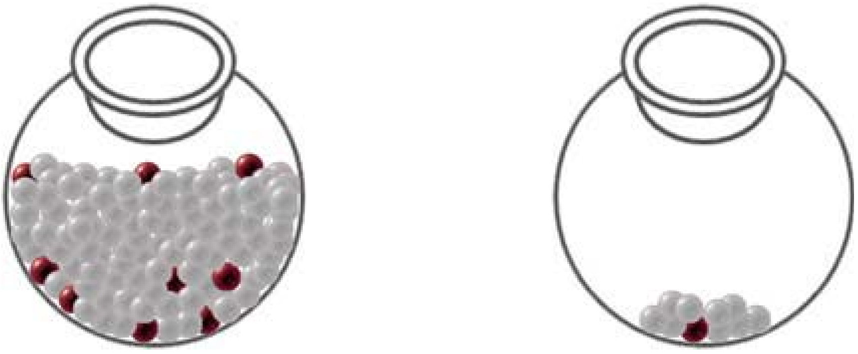

contains 100 marbles, 9 of which are red contains 10 marbles, 1 of which is red

The experimenter will draws a marble from one of the urns. If a red marble is drawn, you win a monetary prize. Which urn do you want the experimenter to draw the marble from?

1. Urn A

2. Urn B

Question 3 - Availability Heuristic (Tversky & Kahneman, 1973) –

Biased response: Selecting instances that are easily remembered:

Let's say you are asked to choose a word at random from an English text. It is more likely that:

1. The word starts with a K
2. K is its third letter

Question 4 - The conjunction fallacy (Tversky & Kahneman, 1983) –

Biased response: evaluating a conjunction event as more probable than a single event:

Linda is 31 years old, single, outspoken, and very bright. She majored in philosophy. As a student, she was deeply concerned with discrimination and social justice issues and participated in anti-nuclear demonstrations. What do you think is more likely?

1. Linda is a bank teller
2. Linda is a bank teller and is active in the feminist movement

Appendix E: Economic scenarios Questionnaire

General instructions: You will be presented with several scenarios taken from everyday life. Please read these scenarios carefully, imagine yourself in that situation, and chose the option that best describes your actions and choices in the same situation. Note that there are no right or wrong answers. So, please choose the option that best describes your preference right now.

Scenario 1 - Outlay for payments:

At the beginning of the new school year, you decide to purchase a TV, a computer, and a refrigerator for your apartment. The total cost is 25,000 NIS. You have the required amount, but since you are a student, you need to consider your future expenses.

The merchant offers you an installment plan with up to 12 equal payments with no interest.

Will you choose to purchase in installments?

A) No
B) Yes

Will your bank account status (credit or debit balance) affect your decision to make the transaction in installments.

A) Yes
B) No

Due to your tight financial situation, you are considering cancelling the order for one of the products. Which product will you decide not to purchase?

A) Television
B) Computer
C) Refrigerator
D) I will purchase all three products

<u>Scenario 2 – Outlay for payments</u>

You have decided to purchase a one-month trip package to the U.S. at the cost of $6,000. Your travel agent allows you to buy the package by credit card in 10 interest-free payments. Your credit card has many pending transactions (although the credit limit has not been exceeded), and you are debating whether to purchase the package on the installment plan. Alternatively, you can take out a loan with interest from the bank and buy the package in one full payment.

Which option do you prefer?

<u>A) Use your credit card to make payments</u>

B) Use a bank loan to pay everything at one time

<u>Scenario 3 - Releasing a savings plan versus taking out a loan</u>

You have had a savings plan of NIS 35,000 in the bank for three years that has the following characteristics :

The interest rate for the 1st year: 0.20%
The interest rate for the 2nd year: 0.65%
The interest rate for the 3rd year: 1.20%

At the end of the first year, you ask the bank to release the money to pay tuition. The bank clerk suggests taking out a loan and not touching your savings plan until the end of the period (the third year) to collect the higher interest rate.

The terms of the loan:
NIS 35,000 for 24 months, with a prime interest minus 0.25% (prime interest is 1.6%).

Which option would you prefer?

A) **<u>Releasing the savings after the first year</u>**

B) Taking out a loan

You can only get one piece of information before taking out a loan. What is the most important piece of information you need to know?

A) Loan amount
B) Duration of the period
C) Interest rate

Appendix F: Lottery Choice Questionnaire

You will be presented with sets of bets. Each bet contains two monetary results expressed as different probabilities (the amounts and probabilities will be displayed on the screen). For each pair, you will be asked to choose the one you would prefer to play. At the end of the experiment, the computer will randomly sample one of your preferred bets, and you will win the monetary amount associated with your response.

	Alternative A	Alternative B
**Pair 1**	**0.8 chance of getting 19.25 NIS, 0.2 chance of getting 0.5 NIS**	0.8 chance of getting 10 NIS, 0.2 chance of getting 8 NIS
**Pair 2**	0.5 chance of getting 10 NIS, 0.5 chance of getting 8 NIS	**0.5 chance of getting 19.25 NIS, 0.5 chance of getting 0.5 NIS**
**Pair 3**	0.6 chance of getting 10 NIS, 0.4 chance of getting 8 NIS	**0.6 chance of getting 19.25 NIS, 0.4 chance of getting 0.5 NIS**
**Pair 4**	0.7 chance of getting 10 NIS, 0.3 chance of getting 8 NIS	**0.7 chance of getting 19.25 NIS, 0.3 chance of getting 0.5 NIS**
**Pair 5**	0.9 chance of getting 10 NIS, 0.1 chance of getting 8 NIS	**0.9 chance of getting 19.25 NIS, 0.1 chance of getting 0.5 NIS**
**Pair 6**	0.4 chance of getting 10 NIS, 0.6 chance of getting 8 NIS	**0.4 chance of getting 19.25 NIS, 0.6 chance of getting 0.5 NIS**
**Pair 7**	**0.1 chance of getting 19.25 NIS, 0.9 chance of getting 0.5 NIS**	0.1 chance of getting 10 NIS, 0.9 chance of getting 8 NIS
**Pair 8**	**0.3 chance of getting 19.25 NIS, 0.7 chance of getting 0.5 NIS**	0.3 chance of getting 10 NIS, 0.7 chance of getting 8 NIS
**Pair 9**	0.2 chance of getting 10 NIS, 0.8 chance of getting 8 NIS	**0.2 chance of getting 19.25 NIS, 0.8 chance of getting 0.5 NIS**
**Pair 10**	**1.0 chance of getting 19.25 NIS, 0 chance of getting 0.5 NIS**	1.0 chance of getting 10 NIS, 0 chance of getting 8 NIS

Appendix G: REI Rational-Experiential Inventory

For each of the following statements, please rate the extent that these items refer to you on a sliding scale ranging from 1 = definitely not true of myself to 5 = definitely true of myself.

	Definitely not true of myself				Definitely true of myself
1. I have a logical mind.	1	2	3	4	5
2. Knowing the answer without having to understand the reasoning behind it is good enough for me.	1	2	3	4	5
3. When it comes to trusting people, I can usually rely on my gut feelings.	1	2	3	4	5
4. I don’t think it is a good idea to rely on one’s intuition for important decisions.	1	2	3	4	5
5. I am not a very analytical thinker.	1	2	3	4	5
6. I prefer complex problems to simple problems.	1	2	3	4	5
7. I suspect my hunches are inaccurate as often as they are accurate.	1	2	3	4	5
8. I often go by my instincts when deciding on a course of action.	1	2	3	4	5
9. I am much better at figuring things out logically than most people.	1	2	3	4	5
10. I trust my initial feelings about people.	1	2	3	4	5
11. I generally don’t depend on my feelings to help me make decisions.	1	2	3	4	5
12. If I were to rely on my gut feelings, I would often make mistakes.	1	2	3	4	5
13. I am not very good at solving problems that require careful logical analysis.	1	2	3	4	5
14. I like to rely on my intuitive impressions.	1	2	3	4	5
15. I enjoy solving problems that require hard thinking.	1	2	3	4	5
16. I believe in trusting my hunches.	1	2	3	4	5
17. I don’t like situations in which I have to rely on intuition.	1	2	3	4	5
18. I don’t like to have to do a lot of thinking.	1	2	3	4	5
19. Using logic usually works well for me in figuring out problems in my life.	1	2	3	4	5

20. I don't have a very good sense of intuition.	1	2	3	4	5
21. I think there are times when one should rely on one's intuition.	1	2	3	4	5
22. I enjoy intellectual challenges.	1	2	3	4	5
23. Reasoning things out carefully is not one of my strong points.	1	2	3	4	5
24. I try to avoid situations that require thinking in depth about something.	1	2	3	4	5

Appendix H: The debt management game

The debt management game:

This section has seven rounds; At the beginning of each round, you will be presented with an amount of money you can use to fully or partially pay off one of two outstanding debts (debt A and debt B), which differ in their initial amount and interest rate.

You must choose which of the two debts you prefer to close and whether you want to invest the full amount of money available to you on each round.

Round 1

**Monetary Amount (NIS) 4,000**		
	**Debt A**	**Debt B**
**Initial Amount (NIS)**	3,000	3,000
**Annual Interest Rate (%)**	2	2.5
**Which of the two debts do you prefer to repay with the amount you received in this round?** Debt A Debt B		

Round 2

**Monetary Amount (NIS) 9,000**		
	**Debt A**	**Debt B**
**Initial Amount (NIS)**	3,000	8,000
**Annual Interest Rate (%)**	2.5	2
**Which of the two debts do you prefer to repay with the amount you received in this round?** Debt A Debt B		

Round 3

**Monetary Amount (NIS) 12,000**		
	**Debt A**	**Debt B**
**Initial Amount (NIS)**	8,000	11,000
**Annual Interest Rate (%)**	2	3.5
**Which of the two debts do you prefer to repay with the amount you received in this round?** Debt A Debt B		

Round 4

**Monetary Amount (NIS) 14,000**		
	**Debt A**	**Debt B**
**Initial Amount (NIS)**	11,000	13,000
**Annual Interest Rate (%)**	3.5	3.25
**Which of the two debts do you prefer to repay with the amount you received in this round?** Debt A Debt B		

Round 5

**Monetary Amount (NIS) 53,000**		
	**Debt A**	**Debt B**
**Initial Amount (NIS)**	13,000	52,000
**Annual Interest Rate (%)**	3.25	3.75
**Which of the two debts do you prefer to repay with the amount you received in this round?** Debt A Debt B		

Round 6

Monetary Amount (NIS) 70,000		
	**Debt A**	**Debt B**
**Initial Amount (NIS)**	52,000	60,000
**Annual Interest Rate (%)**	3.75	4
**Which of the two debts do you prefer to repay with the amount you received in this round?**<br>Debt A Debt B		

Round 7

Monetary Amount (NIS) 70,000		
	**Debt A**	**Debt B**
**Initial Amount (NIS)**	60,000	8,000
**Annual Interest Rate (%)**	4	2
**Which of the two debts do you prefer to repay with the amount you received in this round?**<br>Debt A Debt B		

Appendix I: Repeated savings task

The next section simulates a series of savings account decisions for a period of five years (with quarterly exit points). At the end of each quarter, you are required to choose between two plans.

Your initial amount is NIS 20,000.

The bank clerk asks you to choose between two different savings plans for a period of five years (but you can switch each quarter):

Plan A has a variable interest rate of prime plus 1.5% and plan B a fixed interest rate of 2.5% (details in the table below).

Each plan has a quarterly exit point:

**Monetary Amount (NIS) 20,000**		
	**Plan A**	**Plan B**
**Duration of the plan**	5 years	5 years
**Annual interest at the end of the savings period (%)**	A variable interest of prime plus 1.5	Fixed interest of 2.5

At the end of the first quarter of the first year, the prime interest rate is 1%.

Of the two savings plans, which one do youwant to keep?

Plan A   Plan B

At the end of the second quarter of the first year, the prime interest rate is 1.2%
[No information is given on the prime interest rate]

**Monetary Amount (NIS) 20,000**

	**Plan A**	**Plan B**
**Duration of the plan**	5 years	5 years
**Annual interest at the end of the savings period (%)**	A variable interest of prime plus 1.5	Fixed interest of 2.5

Of the two savings plans, which one do you want to keep?

<u>Plan A</u> Plan B

At the end of the third quarter of the first year, the prime interest rate is 1.4%.

**Monetary Amount (NIS) 20,000**		
	**Plan A**	**Plan B**
**Duration of the plan**	5 years	5 years
**Annual interest at the end of the savings period**	A variable interest of prime plus 1.5	Fixed interest of 2.5

Of the two savings plans, which one do you want to keep?

<u>Plan A</u> Plan B

At the end of the first year, the prime interest rate is 1.6% [No information is given on the prime interest rate]

**Monetary Amount (NIS) 20,000**		
	**Plan A**	**Plan B**
**Duration of the plan**	5 years	5 years
**Annual interest at the end of the savings period (%)**	A variable interest of prime plus 1.5	Fixed interest of 2.5

Of the two savings plans, which one do you want to keep?

<u>Plan A</u> Plan B

At the end of the first quarter of the second year, the prime interest rate is 1.3%.

**Monetary Amount (NIS) 20,000**		
	**Plan A**	**Plan B**
**Duration of the plan**	5 years	5 years
**Annual interest at the end of the savings period (%)**	A variable interest of prime plus 1.5	Fixed interest of 2.5

Of the two savings plans, which one do you want to keep?

<u>Plan A</u> Plan B

At the end of the second quarter of the second year, the prime interest rate is 1.1% [No information is given on the prime interest rate]

**Monetary Amount (NIS) 20,000**		
	**Plan A**	**Plan B**
**Duration of the plan**	5 years	5 years
**Annual interest at the end of the savings period (%)**	A variable interest of prime plus 1.5	Fixed interest of 2.5

Of the two savings plans, which one do you want to keep?

<u>Plan A</u> Plan B

At the end of the third quarter of the second year, the prime interest rate is 0.9%.

**Monetary Amount (NIS) 20,000**

	**Plan A**	**Plan B**
**Duration of the plan**	5 years	5 years
**Annual interest at the end of the savings period (%)**	A variable interest of prime plus 1.5	Fixed interest of 2.5

Of the two savings plans, which one do you want to keep?

Plan A Plan B

At the end of the second year, the prime interest rate is. 0.75% [No information is given on the prime interest rate]

**Monetary Amount (NIS) 20,000**		
	**Plan A**	**Plan B**
**Duration of the plan**	5 years	5 years
**Annual interest at the end of the savings period (%)**	A variable interest of prime plus 1.5	Fixed interest of 2.5

Of the two savings plans, which one do you want to keep?

Plan A Plan B

At the end of the first quarter of the third year, the prime interest rate is 0.85%.

**Monetary Amount (NIS) 20,000**		
	**Plan A**	**Plan B**
**Duration of the plan**	5 years	5 years
**Annual interest at the end of the savings period (%)**	A variable interest of prime plus 1.5	Fixed interest of 2.5

Of the two savings plans, which one do you want to keep?

Plan A Plan B

At the end of the second quarter of the third year, the prime interest rate remains unchanged (0.85%) [No information is given on the prime interest rate]

**Monetary Amount (NIS) 20,000**		
	**Plan A**	**Plan B**
**Duration of the plan**	5 years	5 years
**Annual interest at the end of the savings period (%)**	A variable interest of prime plus 1.5	Fixed interest of 2.5

Of the two savings plans, which one do you want to keep?

Plan A Plan B

At the end of the third quarter of the third year, the prime interest rate is 0.9%.

**Monetary Amount (NIS) 20,000**		
	**Plan A**	**Plan B**
**Duration of the plan**	5 years	5 years
**Annual interest at the end of the savings period (%)**	A variable interest of prime plus 1.5	Fixed interest of 2.5

Of the two savings plans, which one do you want to keep?

Plan A Plan B

At the end of the third year, the prime interest rate is 1% [No information is given on the prime interest rate]

**Monetary Amount (NIS) 20,000**		
	**Plan A**	**Plan B**
**Duration of the plan**	5 years	5 years
**Annual interest at the end of the savings period (%)**	A variable interest of prime plus 1.5	Fixed interest of 2.5

Of the two savings plans, which one do you want to keep?

<u>Plan A</u> <u>Plan B</u>

At the end of the first quarter of the fourth year, the prime interest rate is 1.2%.

**Monetary Amount (NIS) 20,000**		
	**Plan A**	**Plan B**
**Duration of the plan**	5 years	5 years
**Annual interest at the end of the savings period (%)**	A variable interest of prime plus 1.5	Fixed interest of 2.5

Of the two savings plans, which one do you want to keep?

<u>Plan A</u> Plan B

At the end of the second quarter of the fourth year, the prime interest rate remains unchanged (1.2%) [No information is given on the prime interest rate]

**Monetary Amount (NIS) 20,000**		
	**Plan A**	**Plan B**
**Duration of the plan**	5 years	5 years
**Annual interest at the end of the savings period (%)**	A variable interest of prime plus 1.5	Fixed interest of 2.5

Of the two savings plans, which one do you want to keep?

<u>Plan A</u> Plan B

At the end of the third quarter of the fourth year, the prime interest rate is 1.3%.

**Monetary Amount (NIS) 20,000**

	**Plan A**	**Plan B**
**Duration of the plan**	5 years	5 years
**Annual interest at the end of the savings period (%)**	A variable interest of prime plus 1.5	Fixed interest of 2.5

Of the two savings plans, which one do you want to keep?

Plan A Plan B

At the end of the fourth year, the prime interest rate is 1.4% [No information is given on the prime interest rate]

**Monetary Amount (NIS) 20,000**		
	**Plan A**	**Plan B**
**Duration of the plans**	5 years	5 years
**Annual interest at the end of the savings period (%)**	A variable interest of prime plus 1.5	Fixed interest of 2.5

Of the two savings plans, which one do you want to keep?

Plan A Plan B

At the end of the first quarter of the fifth year, the prime interest rate is: 1.5%

**Monetary Amount (NIS) 20,000**		
	**Plan A**	**Plan B**
**Duration of the plan**	5 years	5 years
**Annual interest at the end of the savings period (%)**	A variable interest of prime plus 1.5	Fixed interest of 2.5

Of the two savings plans, which one do you want to keep?

Plan A Plan B

At the end of the second quarter of the fifth year, the prime interest rate is 1.6% [No information is given on the prime interest rate]

**Monetary Amount (NIS) 20,000**		
	**Plan A**	**Plan B**
**Duration of the plans**	5 years	5 years
**Annual interest at the end of the savings period (%)**	A variable interest of prime plus 1.5	Fixed interest of 2.5

Of the two savings plans, which one do you want to keep?

<u>Plan A</u> Plan B

At the end of the third quarter of the fifth year, the prime interest rate is 1.7%.

**Monetary Amount (NIS) 20,000**		
	**Plan A**	**Plan B**
**Duration of the plan**	5 years	5 years
**Annual interest at the end of the savings period (%)**	A variable interest of prime plus 1.5	Fixed interest of 2.5

Of the two savings plans, which one do you want to keep?

<u>Plan A</u> Plan B

At the end of the fifth year, the prime interest rate is 1.8%. [No information is given on the prime interest rate]

**Monetary Amount (NIS) 20,000**		
	**Plan A**	**Plan B**
**Duration of the plans**	5 years	5 years

**Annual interest at the end of the savings period (%)**	A variable interest of prime plus 1.5	Fixed interest of 2.5

Of the two savings plans, which one do you want to keep?

<u>Plan A</u>   Plan B

Table S2 – Repeated Savings Task prime interest summary (Experiment 3)

	1st year				2nd year					3rd year			4th year				5th year			
**Trial number**	1	2	3	4	5	6	7	8	9	10	11	12	13	14	15	16	17	18	19	20
**Prime interest (%)**	1	1.2	1.4	1.6	1.3	1.1	0.9	0.75	0.85	0.85	0.9	1	1.2	1.2	1.3	1.4	1.5	1.6	1.7	1.8
**Rational choice**	A=B	A	A	A	A	A	B	B	B	B	B	A=B	A	A	A	A	A	A	A	A
